# Importance of congruence between communicating and executing implementation programmes: a qualitative study of focus group interviews

**DOI:** 10.1186/s43058-020-00090-w

**Published:** 2020-10-28

**Authors:** Lars H. Lindholm, Minna Laitila, Antero Lassila, Olli Kampman

**Affiliations:** 1 Department of Psychiatry, South Ostrobothnia Hospital District, Hanneksenrinne 7, FI-60220 Seinäjoki, Finland; 2 Faculty of Medicine and Health Technology, Tampere University, PO Box 100, FI-33014 Tampere, Finland; 3 Department of Psychiatry, Pirkanmaa Hospital District, PO Box 2000, FI-33521 Tampere, Finland

**Keywords:** Evidence-based treatment, Implementation, Programme evaluation, Effectiveness-implementation hybrid design, Normalization Process Theory, Focus group interview

## Abstract

**Background:**

The Ostrobothnia Depression Programme (ODP) in Finland was intended to implement two evidence-based brief psychotherapy interventions, namely motivational interview and behavioural activation, in several regional psychiatric teams. A simultaneous effectiveness study was conducted. Considerable tension was encountered between these two arms, causing resistance to change. We conducted a qualitative case study to better understand this tension and to discuss how managerial and executive practices may ensure the successful running of a hybrid design programme.

**Methods:**

We conducted focus group interviews to evaluate the phases of preparation and practical execution of the ODP from the perspectives of management and the programme executives. To gather the data, we applied the revised Socratic approach for health technology assessment and focus group interviews. We analysed the data deductively according to the Normalization Process Theory.

**Results:**

We identified two main critical issues: (1) The ODP programme plan ignored the team leaders’ crucial role in influencing the implementation climate and mobilizing organizational strategies. The ODP had a simplistic top-down design with minimal and delayed collaboration with its target groups in the preparation phase. (2) Incongruence occurred between what the project group had explicitly communicated about being the spearhead of the ODP and what they then actually enacted. These two issues caused tension between the implementation efforts and the effectiveness study as well as resistance to change among the staff.

**Conclusion:**

Early, open collaboration with all prospective stakeholders towards a shared understanding about the programme is the first action the programme administrators should take. Agreement on goals and the means to achieve them would lower tension between the two arms of a hybrid design programme, thereby reducing resistance to change. Congruence between the goals communicated and the actual managerial and executive actions is of paramount importance in getting the programme recipients on board.

**Supplementary Information:**

The online version contains supplementary material available at 10.1186/s43058-020-00090-w.

Contributions to the literature

- Our results reveal a risk of tension between the simultaneous implementation efforts and the effectiveness study.
- It is important to systematically maintain the balance determined between the two arms of a hybrid design programme.
- We identified two contrasting ways of responding to the same programme and explain and discuss their implications.
- We contribute to what is known about the need for early collaboration with every stakeholder group of a programme to motivate their readiness for change.
- We found the Revised Socratic Approach for Health Technology Assessment a feasible instrument, also for assessing immaterial heath technologies.

## Background

Quality improvement is the main aim of a programme for implementing an evidence-based treatment (EBT) in the context of a health care organization [1]. The ultimate intended beneficiaries are the patients. The key challenge for programme administrators is to develop a programme plan encouraging frontline treatment providers to incorporate the EBT into their routine practices [2]. Several theory-based implementation models or frameworks were constructed to facilitate the work [3–9].

Several determinants for the acceptance of an implementation programme have been identified [5, 10]. These include top-down vs. bottom-up programme design, early vs. late collaboration with each stakeholder group, and the leaders’ reactions to various manifestations of readiness for change among the relevant personnel [11–13]. The role and performance of leadership have been reported to be critical for the success of a programme and also for sustaining its outcomes [2, 14–16]. The factors above, in turn, have an influence on the implementation climate, by which we mean the shared receptivity of the staff involved [5]. ‘Programme theory’ is a concept that refers to an individual idea about what might be achieved and by which interventions or operations in a given context [17]. This theory, in turn, guides those responsible for the programme in designing the programme plan. They may accomplish this work either heuristically, relying on their previous experience and expertise, or then methodically, grounding their design in a theory-based framework or model, or then a combination of these [7].

Ensuring that an intervention continues to be effective throughout an implementation programme is a fundamental concern [18]. Conducting effectiveness-implementation hybrid design studies is a rising and much advocated approach to address this concern [19, 20]. In such a study, these two arms run concurrently. Hybrid design studies are likely to expose the potential tensions inherent in real-world implementation processes of EBTs and their impacts on their application [21, 22]. For instance, some elements of the original intervention may require adjustment to the real-world setting, thereby risking impaired efficacy [21]. This lends support to the call for increasing the application of hybrid designs to gather more clinically quality-controlled knowledge on implementation efforts [23, 24]. However, not enough is known about possible procedural tensions between effectiveness studies and implementation efforts in naturalistic settings and this gap needs to be addressed.

The administration of the psychiatric department of South Ostrobothnia Hospital District in Finland launched the Ostrobothnia Depression Programme (ODP) [25]. The main goal was to bring about a change in the clinical practices to bridge the gap between the resources available and the increasing demand for treatment for depressive patients. The ODP carried out both the implementation programme and the effectiveness study for two evidence-based brief psychotherapy interventions, namely motivational interviewing (MI) and behavioural activation (BA) [26, 27]. A quantitative evaluation of the implementation programme showed that a third of the target group were active adopters of MI and BA [28]. The effectiveness study yielded positive results [29].

Earlier evaluations of the ODP implementation were conducted among the frontline therapists, the intended adopters of MI and BA. In the summative evaluation, the implementation outcome only reached a third of the target group. It also revealed that the ODP lacked strategies for sustaining and scaling up the implementation outcomes in the long term [28]. This was attributed in part to the weak role of the team leaders in the programme execution and was strengthened in the mixed-methods evaluation of the influence of different organizational- and programme-related factors (Lindholm et al., submitted). In addition, considerable resistance to change was encountered in some of the participating teams while others welcomed the ODP. These observations led us to augment the overall evaluation qualitatively with a special focus on the managerial and executive processes. We hypothesized that these social processes in designing and executing the ODP would explain the tension related to conducting the hybrid design programme. This case study was to test our hypothesis. We also discuss how the information obtained could be considered on future programmes.

### The ODP

**Fig. 1:**
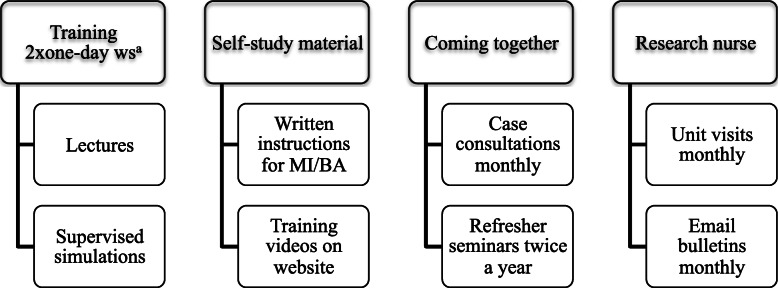
Programme resources allocated to the Ostrobothnia Depression Programme. Attending training was the only prerequisite for a therapist to be regarded as ODP enrolled. Superscript letter ‘a’ indicates 1-day training workshops for both behavioural activation and motivational interviewing

The hospital district in charge of the ODP is responsible for the provision of public specialized health care services to a population of 200,000. The ODP ran during the period 2009–2013. It was a regional programme comprising two integrated subprogrammes: the Ostrobothnia Depression Study (ODS) and related Implementation Programme (ODS-I). The ODP was aimed to encourage frontline therapists to implement MI and BA and to recruit patients for an effectiveness study of these interventions. Thus, the ODP had a hybrid effectiveness-implementation design, although the term was not used as the ODP was launched a few years before the term was introduced [19]. Participation in the training in MI and BA and in applying them in everyday work did not constitute commitment to recruiting patients for the effectiveness study. However, this was intensely encouraged. The programme resources are presented in Fig. 1 and the therapists’ tasks regarding the effectiveness study in Table 1.

**Table 1:** Tasks for a therapist regarding the Ostrobothnia Depression Programme-related effectiveness study. The doctors were responsible for diagnostics and medication. Patients referred to psychiatric secondary services because of depressive symptoms, anxiety, self-destructiveness, insomnia and alcohol or other substance-related problems were screened for recruitment. The inclusion criterion was 17 points in Beck Depression Inventory, 21-item (BDI-21), but patients with psychotic disorders or organic brain disease were excluded

Patient’s first visit to a unit/therapist	Recruitment - Giving information about the study - Requesting a written informed consent After the consent - Filling in a three-page patient data form - Performing a structured patient assessment (BDI-21, AUDIT, alcohol dose counter form, GAF, MINI-C) - Filling in a referral to laboratory tests
During the treatment	Every 2 weeks - BDI-21 When necessary - CIWA-Ar - Patient follow-up form after detoxification - Study discontinuation form

*AUDIT*, Alcohol Use Disorder Identification; *GAF*, Global Assessment of Functioning; *MINI-C*, Mini International Neuropsychiatric Interview, module C for assessment of suicidality; *CIWA-Ar*, Clinical Institute Withdrawal Assessment for Alcohol Scale

The goal to develop clinical practices to meet the increasing flow of depressive patients was initiated by the hospital district administration. The clinical director of the psychiatric department assembled a project group to elaborate a programme for this purpose. In addition to the clinical director (MD, PhD), the project group comprised principal and associate programme executives (a professor of psychiatry and a registered psychologist, respectively) and a senior consultant (MD, PhD), all of them permanently employed in the hospital district. They deemed an effectiveness study important for the quality control of the innovation. For the execution, the project group was reinforced with two assisting research nurses.

## Methods

The unit external to the ODP managing organization was excluded from the present study in order to focus the evaluation on intra-organizational processes. We collected the present data in March 2015, 16 months after the end of the ODP. The time gap was because the analyses of the final summative inquiry and mixed-methods study, both administered to the frontline therapists in spring 2014, revealed a need to complement our understanding about administering of the ODP. The present authors’ connections to the ODP and the present study are presented in Table 2. Also, their connections to the managing organization and their mutual professional relationships are presented in Additional File 1. In reporting the study, we adhered to the 32-item checklist of consolidated criteria for reporting qualitative studies (COREQ), which is presented in Additional File 2 [30].

**Table 2:** Participants’ various relations to the study and manuscript

Relation to the study	Role in the interviews	Relation to drawing the final results	Relation to the manuscript
Main researcher	Interviewer	Main	First author
Collaborating researcher	Secretary^a^	Collaborating	Second author
Study group			
*Focus group 1/the project group, five members*			
Clinical director of the psychiatric department	Informant	Acceptance	Third author
Principal designer and executive of the programme, prof. in psychiatry	Informant	Acceptance	Fourth author
Associate designer and executive, reg. psychologist	Informant	Acceptance	None
Two assisting research nurses	Informants	Acceptance	None
*Focus group 2/team leaders, eight members*			
Eight people, both psychiatrists and registered nurse	Informants	None	None

^a^The collaborating researcher had to be excluded from the interview of focus group 2 due to her managerial relation to its nurse members, so the main researcher also took notes while interviewing. The notes were checked afterwards against the videotapes

### Setting

#### Formation and description of the study group

We assembled the study group according to the purposeful sampling strategy ‘complete target population’ [31]. We emailed the invitation to the whole ODP project group and all team leaders of the target units, 14 individuals in total. Only one recipient, involved in the project group, declined the invitation due to compelling personal reasons, thus resulting in a study group of 13 individuals. We informed the study group in advance about the purpose, setting and course of the study as well as the principles for handling the data. This included information about the videotaping of the interviews and the assurance that no interviews would be transcribed due to the sensitive nature of the material and further the assurance that each participant’s identity would be protected as far as possible during processing and utilization of the information obtained. Recipients were assured that participation in the study was voluntary and would in no way affect their status within the organization. All members of the study group gave verbal consent to participate.

The study group was divided into two focus groups (FG 1 and FG 2) according to each member’s relation to the ODP: FG 1 comprised the project group and FG 2 team leaders (for more detail, see Table 2). All members of the study group and the researchers had been permanently employed in the organization for years before the launching of the ODP; thus, their relationship was established prior to the present evaluation.

#### Interview protocol and guides

**Fig. 2:**
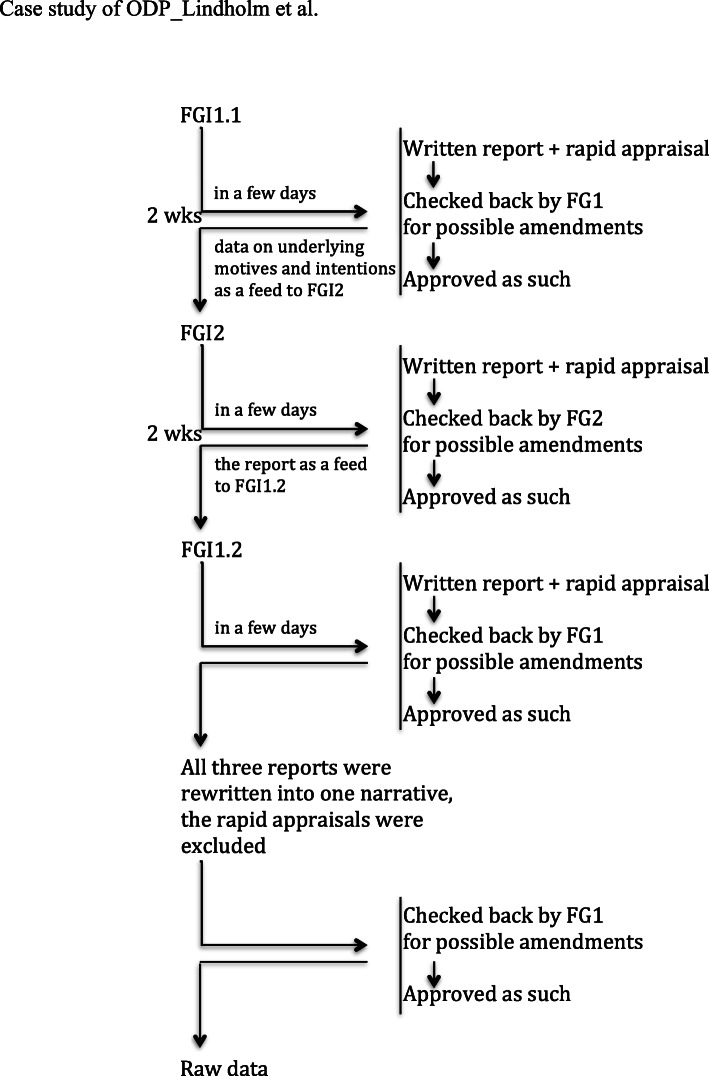
Setting for the iterative semi-structured focus group interviews and gathering the raw data. Abbreviations: FG 1 focus group 1, FG 2 focus group 2, FGI 1.1 the first interview with FG 1, FGI 2 interview with FG 2, FGI 1.2 second interview with FG 1

We interviewed FG 1 twice (FGI 1.1 and FGI 1.2) and FG 2 once (FGI 2) (Fig. 2). The iterated interview with FG 1 was to involve the project group reflexively in appraising the data obtained so far and completing the description of ODP processes. This was done to ensure richer and more accurate data. Each interview lasted 3 h and was divided into two parts with a short break between them. Four members out of five in FG 1 and five out of eight in FG 2 attended the group interviews in person. Four individuals were unable to attend the group interviews in person due to pressure of work but provided the desired information in alternative ways: The FG 1-enrolled associate executive was interviewed separately immediately after FGI 1.1, and the information obtained was included in the respective report. One FG 2-enrolled person provided written feedback before FGI 2, and this information was presented to FG 2 during the interview. The remaining two FG 2-enrolled people who were unable to attend in person had discussed the issues beforehand with their attending colleague.

We compiled two interview guides: the first for the interviews with FGI 1.1 and FGI 2 and the second for the interview with FGI 1.2. The first interview guide covered five topics that we considered to require in-depth evaluation. These topics were the underlying motives and intentions of the ODP, its management, the perspectives of the participating units and the interests of the individuals conducting the present evaluation and creating a quick vision for future developments. The actual questions to be asked during the interview were selected and adapted from the Revised Socratic Approach for Health Technology Assessment [32]. This approach is presented more specifically in Additional File 3, and the creation of the first interview guide is presented in more detail in Additional File 4, Table A. The second guide, for the interview with FGI 1.2, was composed to ensure rich and valid data in collaboration with those responsible for the ODP (see Additional File 4, Table B).

While creating the interview protocol and guides, the first author had reflective discussions about the mission and procedure with the collaborating researcher, the clinical director and the principal programme executive. Due to the setting, we had no opportunity to pilot the interview protocol and guides in practice.

#### Forming the raw data

As a base, we had the technical data on the ODP comprising the implementation plan of ODS-I [28], the research plan of the ODS including the protocol for data collection (Table 1) and total executive resources in ODP (Fig. 1). We gathered the supplementary information through an iterative and collaborative process in the FGIs. Finally, we wrote one, rich narrative on administering the ODP, which served as the raw data. See more detail in Fig. 2.

### Qualitative content analysis

The case of our study was the process of running the ODP all the way from its rationales to its completion, and the unit of analysis was the narrative that served as the raw data [31]. We analysed the raw data through deductive qualitative content analysis [33] guided by Normalization Process Theory (NPT) [34, 35]. The NPT is presented more specifically in Additional File 3 and the coding frame in Additional File 5. Our analysis and extracting the results progressed in four steps: First, we encoded the raw data using different colours and reorganized it according to the main categories. Second, we re-encoded and organized the data further according to the subcategories. We reviewed the relevance of the encoding during the two first steps and readjusted when needed. Third, we condensed and rewrote the information contained in the encoded text pieces into a fluent narrative in terms of each subcategory. Fourth, we extracted the relevant information in terms of our hypothesis from the data analysed, thereby providing the results of the present study.

The first author performed the coding and extracted the results in close consultation with the second author. Finally, we presented the results to the members of FG1 for appraisal and possible amendments. They suggested some refinements and, after these had been made, they accepted the results presented below. The analysis of the data was processed manually with assistance of Word for Mac 2011.

## Results

**Fig. 3:**
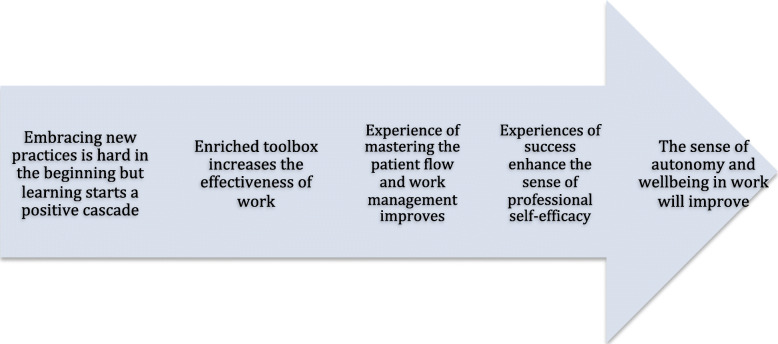
Presumed cascade of the impacts of the Ostrobothnia Depression Programme at the level of the individual therapist

Two main critical issues emerged, which we interpreted to shed light on the friction encountered during the ODP: (1) The programme theory was grounded on the conception that the goals of the ODP were feasible by addressing programme strategies almost exclusively to frontline therapists (Fig. 1). To the frontline therapists focusing strategy was based on the idea of learning by doing. The programme theory was purely heuristic and implicit and was not tested against any formal implementation theory or model. Those who designed the ODP drew on their previous experience of administering developmental programmes and also on their pedagogical expertise and experience of serving as trainers. In addition, they had individual experience of their own training in psychotherapy having a positive impact on mastering clinical work (Fig. 3). (2) Right from the outset, there was tension between the simultaneously administered implementation programme and the effectiveness study. Although the programme executives explicitly articulated that implementation and quality improvement were the primary intention, the ambitions related to the effectiveness study practically outstripped those of the implementation programme. The results underlying these two issues are next presented in more detail.

### Lack of involvement of key stakeholders

The reasoning and determination of the ODP goals were constructed mostly at a high level in the organization (Table 3). Prior to the ODP, the clinical director was aware of increasing distress among the frontline personnel due to accelerating patient flow, and the ODP was launched to tackle the problem. The project group included no lower level leaders or frontline therapists from the intended target group. The team leaders were not invited until the phase of finalizing the programme plan. The primary goal the project group had set for the ODP was to achieve quality improvement in clinical practices. However, in the experience of the team leaders, the preparatory process had proceeded one-way, top-down, which they considered to be a deviation from the normal collaborative two-way managerial practices adhered to while preparing organizational strategies. They saw one-way preparation as a normal and acceptable practice for research programmes. In addition, in the name of the programme, the term ‘study’ preceded the term ‘implementation’, which, they said, strengthened their perception that the research was accorded priority. In summary, the specification of the ODP was not a collaborative effort between various stakeholders, who thus achieved no shared understanding about the emphasis between the two endeavours.

**Table 3:** Preparation of the Ostrobothnia Depression Programme (ODP) and stages of involving different stakeholder groups

Stakeholder	Stage		Description
The project group^a^	I	Identifying and analysing the problem to be tackled	Obstructed patient flow and difficulties in work management.
	II	Defining the goals	1. Speed up the treatment process by increasing delivery of brief therapies 2. Increase application of the integrated treatment model to make up for the deficit in the treatment of dual diagnosis patients 3. Measure the effectiveness of the treatment model 4. Improve the work well-being of the staff by strengthening their work management
	III	Preparation of the programme plan	a. Determining the criteria for selecting the interventions to implement b. Determining the criteria for inviting the units to participate c. Designing the treatment model d. Designing the implementation plan e. Designing the protocol for the effectiveness study
The project group and team leaders	IV	Finishing the programme plan	a. The project group consulted the team leaders a few times for amendments b. The plan was modified slightly in terms of practical execution according to the comments

^a^The project group = the clinical director of the department of psychiatry, the principal and associate executives of the ODP and a senior consultant, and for execution, the group was reinforced with two assisting research nurses. They were all permanently employed in the main target organization

### Deficient consideration for readiness for change in recruitment efforts

Participation in the ODP was originally voluntary for the units invited, at least in principle. Not all psychiatric units of the hospital district were invited. The invitations were targeted according to two criteria: (1) the clinical director’s impression of the positive readiness for change in the units and (2) the number of patients needed for the effectiveness study. The largest unit was invited according to the second criterion only, that is, to satisfy the needs of the research. Since the largest unit initially declined, they were persuaded to participate after a 1-year delay. The other units accepted the invitation at the first step. In summary, involving the largest unit in the ODP was fundamentally incongruent with the first invitation criterion, i.e. readiness for change, and actual willingness to participate on the part of the staff.

### Absence of buy-in among key stakeholders

Most of the voluntarily participating units’ team leaders saw the ODP as an opportunity to learn something new and to review the prevailing treatment practices, although they still saw it primarily as a research programme. The reception of the ODP between units varied from welcoming it, through confusion, to considerable resistance. The programme executives identified one team where the collaboration had been smoothest. The ideas in the ODP were congruent with the team’s own ideas, which they had already been working with. By contrast, considerable resistance arose in the largest unit, which had initially been reluctant to participate. In addition, a previously unarticulated confusion about the ultimate intentions of the ODP was eventually articulated. Tackling the resistance greatly depleted the executives’ resources. The team leader of this unit deemed the goals for the ODP to be relevant but considered that the change aimed at was too ambitious to be loaded on one programme. Moreover, the team leader appraised merging programmes for implementation and clinical research as an improper setting to reach the goals.

Despite enrolment in the ODP at the level of units, the enrolment of the therapists in the training varied widely between the units. At best, all the therapists of one unit completed the training. At worst, only one or two therapists of a unit joined in, including one temporary substitute. Some of the units assigned more therapists to the training later on and some of them were motivated mainly by the hope of simply getting the ODP over and done with. In summary, despite the participation, collective inclination to work on the ODP varied widely across units between adherence and resistance. Two essential manifestations of this tension were identified: the therapists’ enrolment in the training varied across units from poor to comprehensive and the motivation of some therapists joining at a later stage was dubious.

### Participant withdrawal and turnover

Due to at least two reasons, the number of patients to be recruited for the effectiveness study was accumulated more slowly than anticipated: (1) some of the initially keen therapists got tired in the course of the programme and withdrew and (2) staff turnover cut down the number of ODP-trained therapists. Recruiting patients began to accumulate on fewer shoulders, which caused stress. The question, ‘when will this be over?’ arose among the therapists. In summary, the accumulation of workload biased progressively as the ODP proceeded, resulting in programme fatigue.

### Failure to focus on implementation effort

Some positive experiences in the early phase encouraged the programme executives to think that the strategies applied in the ODP had the potential to bring about the desired cultural change in treatment practices at the level of the entire department. However, they became hesitant as the programme proceeded, partly because they noticed that patient recruitment for the effectiveness study occupied too large a role and the idea of implementation faded. The team leaders had a shared perception that the concurrent running of the implementation and clinical research programmes caused confusion among the therapists. The number of patients needed for the effectiveness study was intended not only to ensure the strength of the study but also a sufficient amount of practice needed to consolidate skills in BA and MI. The drive to satisfy the scientific interest escalated as the ODP proceeded, and this exacerbated the therapists’ sense of pressure, which further increased their negative perception of the ODP. In summary, enthusiasm for the implementation declined and the effectiveness study gained in significance as the ODP progressed, which jeopardized achieving the original goal of extensive implementation of BA and MI.

## Discussion

Our analysis revealed two key factors and related phenomena, which helped to understand the course of the ODP: (1) The programme theory. This was based on the project group’s experience of previous developmental programmes and expertise in training. In addition, they assumed that the ODP goals would be feasible by addressing the programme strategies almost exclusively to the frontline therapists. This assumption in particular led to a too narrow programme theory, which ignored the team leaders’ crucial role in influencing the implementation climate and mobilizing organizational strategies [2, 15]. (2) Coherence between what was explicitly communicated and what was practically accomplished on a programme. The ODP was communicated primarily as an implementation programme for EBTs. However, the target teams perceived that research was prioritized. These two main findings establish our hypothesis that practices of administering the ODP laid the programme open to tension between the implementation efforts and the effectiveness study encountered right from the beginning. However, the results provided us with two entry points to discuss the preferable measures of the managerial and executive practices enabling a hybrid design programme and overcoming resistance to change.

A programme theory is an individual compilation of beliefs as to what a programme might achieve and by what means [17]. These beliefs determine the practical actions that the programme administrators will take. While building an evidence-based programme theory, the heuristic ideas are tested and complemented according to some appropriate framework or model [7]. In an optimal case, the programme theory will be resilient and elaborated in early collaboration with the intended programme addressees [17]. Contrary to this, the ODP programme theory was built heuristically only and at a high organizational level and the team leaders were only brought in at a later stage. The Consolidated Framework for Implementation Research (CFIR) is a determinant framework that provides a panel of 39 evidence-based factors, disposed under five domains, impacting the success of an innovation implementation [5, 36]. Reflected against the CFIR, the ODP programme theory ignored the determinants of ‘tension for change’, ‘learning climate’ and ‘leadership engagement’. Taking these into account would have induced early collaboration with both team leaders and frontline therapists to pursue a communal specification of the ODP. Indeed, early collaboration may initially cause the start-up of a programme to be more complex and time-consuming and, consequently, require more resources. However, such investments may be recouped later on in terms of less resistance and smoother-running programme [2, 14, 37].

Coherence in communication and executive actions is of paramount importance. Regarding the ODP, the fundamental lack of coherence emerged in terms of the question of the primary goal. This is closely related to the lack of early collaboration between different stakeholders reported in the previous paragraph. The ODP was communicated as being essentially an EBT implementation programme. However, the mode of preparation caused the target teams to regard it primarily as an effectiveness study. Inducing the largest unit to participate primarily to ensure the strength of the effectiveness study and ignoring the first criterion of inviting the units, i.e. the readiness for change, conveyed a non-verbal message inconsistent with what had initially been articulated. Moreover, towards the end of the ODP, satisfying the patient count needed for the effectiveness study over-rode the idea of implementation. These phenomena also caused and exacerbated misunderstandings between the various stakeholders. In spite of this, one team found the ODP to be consistent with their own developmental efforts in the past, which was also apparent in their positive readiness for change. This led them to the conclusion that the ODP provided them with an opportunity to improve their professional capability, which, in turn, supported their cognitive participation in the ODP [34]. The negative experience arose from the conviction that connecting the implementation of two EBTs and their effectiveness study was too much, which exacerbated an already unreceptive climate. This fuelled the perception that the two arms of the ODP were in competition with each other, which culminated in a sense of administrative pressure. Furthermore, this caused frustration and rejection among the staff, which can be seen as negative manifestations of cognitive participation and collective action according to NPT [34].

Studying the effectiveness of an EBT in connection with its implementation programme serves as a clinical quality control and ensures movement in the right direction [1, 10, 18, 21]. This was also one reason for the hybrid design in the ODP. Additionally, the effectiveness study was assumed to prompt the therapists to actively adopt the EBTs and thus ensure the accumulation of a sufficient amount of clinical practice for acquiring skills in the EBTs. Consequently, in principle, the implementation programme had priority. However, the ODP was inherently contradictory in terms of the priority order of the two objectives loaded on it, which caused confusion. Such a situation was likely to lead to a perception that the different objectives were actually competing against each other [10]. Adjusting the ODP as a whole with respect to the teams’ varying reactions regarding the implementation climate would also have entailed adjustments in administering the effectiveness study.

### Strengths and limitations

We reached all but one out of the intended informants since we accepted other ways of providing information than only individual attendance at the FGIs, which ensured obtaining a wide range of opinions. On the other hand, one more iteration with both FGs and inviting a third focus group from the frontline therapists would have provided us with richer data. Also, in not transcribing the interviews, we deviated from the conduct of the conventional qualitative interview study. We made this decision as we were interested in the data verbally articulated, not the non-verbal data. These restrictions enabled us to keep the research process within our resources. In spite of these limitations, we consider that we reached our goal to scrutinize the social processes related to the ODP and thus identify the risks inherent in conducting an effectiveness study in connection with an implementation programme. We state that the NPT was an appropriate tool for the purpose. In addition, we extended the existing knowledge about the need to ensure early collaboration with every stakeholder group.

### Fidelity of the data

Special attention was paid to the general atmosphere during the FGIs and to ensuring that the data articulated on the questions of interest was clearly expressed [38]. During the interviews, a free and frank dialogue was achieved, where both disagreement and consensus within and between the groups were accepted. A report on each FGI was written only a few days after the interview and sent for confirmation to each participant in the FGI concerned. All reports were approved as such. In addition, the facilitator checked the reports by watching the videotaped interviews and no new substantive information was detected although some amplificatory and descriptive details, e.g. quotations, were indeed picked up. The foregoing serves to verify the true correspondence between the essential contents of the FGIs and the raw data. In addition, the members of the FG1 reviewed the present results section, which was amended according to the feedback.

## Conclusion

Early, open collaboration with all intended stakeholders for pursuing a communal specification, i.e. a shared understanding, about the programme is the first action programme administrators should take on launching an EBT implementation programme. This has a direct link to the programme theory about what the programme has the potential to reach and how. Early collaboration would have improved the mutual understanding among the stakeholders and helped the administrators to take all relevant aspects into account. Congruence between what the programme administrators communicate and what they actually do is the second thing to be strictly adhered to throughout the programme. This is crucial to avoid confusion regarding the ultimate mission of the programme. Hybrid design programmes have the potential to achieve quality-controlled outcomes in implementing health care innovations or reforms. However, they require careful attention to keeping the balance consistent between the programme’s primary mission and the effectiveness study. This and early collaboration are principles the clinical managers and programme executives should adopt to enable the implementation of health care innovations or reforms and to overcome resistance to change.

## Supplementary information

**Additional file 1.** The authors’ professional relations to ODP, the organisation and each other.

**Additional file 2.** The 32-item checklist of consolidated criteria for reporting qualitative studies (COREQ).

**Additional file 3.** Descriptions and rationale behind of two instruments.

**Additional file 4.** The interview guide for the first interviews of Focus Groups 1 and 2 and the process of deriving of the guide (Table A). The interview guide for the second interview of Focus Group 1 (Table B).

**Additional file 5.** Coding frame according to Normalization Process Theory.

## Data Availability

The original datasets generated and analysed during the present study are not publicly available due to the requirement to preserve confidentiality. However, the final narrative about the OPD, that is the raw data, is available from the corresponding author on reasonable request. The raw data is in Finnish. We made the English translation during the fourth step of the analysis, i.e. when extracting the results.

## Abbreviations

BA: Behavioural activation
COREQ: 32-item checklist of consolidated criteria for reporting qualitative studies
EBT: Evidence-based treatment
FG: Focus group
FGI: Focus group interview
MI: Motivational interviewing
NPT: Normalization Process Theory
ODP: Ostrobothnia Depression Programme
ODS: Ostrobothnia Depression Study
ODS-I: Ostrobothnia Depression Study related Implementation Programme
FG 1: Focus group 1
FG 2: Focus group 2
FGI 1.1: First interview with FG 1
FGI 1.2: Second interview with FG 1
FGI 2: Interview with FG 2
