# Supplementary material for: Importance of congruence between communicating and executing implementation programmes: a qualitative study of focus group interviews
Source: Implement Sci Commun. 2020 Oct 28;1:94. doi: 10.1186/s43058-020-00090-w (PMC7594330; doi:10.1186/s43058-020-00090-w)
Supplement: Supplementary file 1 — Additional file 1. The authors’ professional relations to ODP, the organisation and each other. [file 43058_2020_90_MOESM1_ESM.pdf]

**The authors' professional relations to ODP, the organisation and each other**

The first and second authors, LHL and ML, were not involved in the design or execution of the ODP. LHL is the main researcher of the present study. He is an MD, specialist in psychiatry and a PhD student. He had formerly made a career as a general practitioner in a public health care centre and within occupational health care. During those years, he became interested in organizational psychology and in the management of organizational change. ML is a collaborating researcher. She is a registered nurse and holds a PhD in nursing science. During the ODP, she was employed as a nursing director on the units engaged in the programme. Her everyday work involved close collaboration with the clinical director of the psychiatric department.

The third and fourth authors, AL and OK, were both closely involved in the design and execution of the ODP. AL is the clinical head of the psychiatric department. He was the manager responsible for the ODP and also a participant in FG1 as an interviewee. OK is a professor of psychiatry. He is employed both in Tampere University and Ostrobothnia Hospital District. OK was the principal designer and programme executive of ODP and also a participant in FG1 as an interviewee.

The first author, LHL collaborates closely with AL and OK.  In his everyday clinical work, LHL is directly subordinate to AL. In turn, OK was the main trainer of LHL during his residency in psychiatry and OK is also the supervisor of LHL's doctoral research, of which the present study forms part. We have identified the reflexive risks that the depicted relationships pose to the reliability of the study. Therefore, we have had several open conversations about the issue in an attempt to mitigate that risk.
