# Supplementary material for: Importance of congruence between communicating and executing implementation programmes: a qualitative study of focus group interviews
Source: Implement Sci Commun. 2020 Oct 28;1:94. doi: 10.1186/s43058-020-00090-w (PMC7594330; doi:10.1186/s43058-020-00090-w)
Supplement: Supplementary file 2 — Additional file 2. The 32-item checklist of consolidated criteria for reporting qualitative studies (COREQ). [file 43058_2020_90_MOESM2_ESM.pdf]

Consolidated criteria for reporting qualitative studies (COREQ): a 32-item checklist

Adopted form Tong A, Sainsbury P, Craig J. Consolidated criteria for reporting qualitative research: A 32-item checklist for interviews and focus groups. Int J Qual Heal Care. 2007;19(6):349–57.

No Item	Guide questions/description	Reported on page
**DOMAIN 1: RESEARCH TEAM AND REFLEXIVITY**		
**Personal Characteristics**		
1. Interviewer/facilitator	Which author/s conducted the interview or focus group?	7 (Table 2)
2. Credentials	What were the researcher's credentials? *E.g. PhD, MD*	Additional File 1
3. Occupation	What was their occupation at the time of the study?	8 (Setting)
4. Gender	Was the researcher male or female?	Additional File 1
5. Experience and training	What experience or training did the researcher have?	Additional File 1
**Relationship with participants**		
6. Relationship established	Was a relationship established prior to study commencement?	8 (Setting)
7. Participant knowledge of the interviewer	What did the participants know about the researcher? *e.g. personal goals, reasons for doing the research*	8 (Setting), Additional file 1
8. Interviewer characteristics	What characteristics were reported about the interviewer/facilitator? *e.g. bias, assumptions, reasons and interests in the research topic*	Additional File 1
**DOMAIN 2: STUDY DESIGN**		
**Theoretical framework**		
9. Methodological orientation and Theory	What methodological orientation was stated to underpin the study? *e.g. grounded theory,*	9-10 (Methods), Additional File 3

	discourse analysis, ethnography, phenomenology, content analysis	
**Participant selection**		
10. Sampling	How were participants selected? *e.g. purposive, convenience, consecutive, snowball*	8 (Setting)
11. Method of approach	How were participants approached? *e.g. face-to-face, telephone, mail, email*	8 (Setting)
12. Sample size	How many participants were in the study?	8 (Setting)
13. Non-participation	How many people refused to participate or dropped out? Reasons?	8-9 (Setting)
**Setting**		
14. Setting of data collection	Where was the data collected? *e.g. home, clinic, workplace*	N/A
15. Presence of non-participants	Was anyone else present besides the participants and researchers?	N/A
16. Description of sample	What are the important characteristics of the sample? *e.g. demographic data, date*	7 (Table 2),<br>8 (Setting)
**Data collection**		
17. Interview guide	Were questions, prompts, guides provided by the authors? Was it pilot tested?	7 (Table 2)<br>10 (Setting),
18. Repeat interviews	Were repeat interviews carried out? If yes, how many?	8 (Setting),<br>9 (Figure 2)
19. Audio/visual recording	Did the research use audio or visual recording to collect the data?	8 (Setting),<br>18 (Fidelity of the data)
20. Field notes	Were field notes made during and/or after the interview or focus group?	7 (Setting)
21. Duration	What was the duration of the interviews or focus group?	8 (Setting)

22. Data saturation	Was data saturation discussed?	17 (Strengths and limitations)
23. Transcripts returned	Were transcripts returned to participants for comment and/or correction?	9 (Figure 2), 18 (Fidelity of the data)
**DOMAIN 3: ANALYSIS AND FINDINGS**		
**Data analysis**		
24. Number of data coders	How many data coders coded the data?	10 (Qualitative content analysis)
25. Description of the coding tree	Did authors provide a description of the coding tree?	Additional File 5
26. Derivation of themes	Were themes identified in advance or derived from the data?	9 (Setting)
27. Software	What software, if applicable, was used to manage the data?	11 (Qualitative content analysis)
28. Participant checking	Did participants provide feedback on the findings?	9 (Figure 2), 18 (Fidelity of the data)
**Reporting**		
29. Quotations presented	Were participant quotations presented to illustrate the themes/findings? Was each quotation identified? *e.g. participant number*	N/A
30. Data and findings consistent	Was there consistency between the data presented and the findings?	10-11 (Qualitative content analysis)
31. Clarity of major themes	Were major themes clearly presented in the findings?	11-14 (Results)
32. Clarity of minor themes	Is there a description of diverse cases or discussion of minor themes?	11-14 (Results), 16-17 (Discussion)
