# Supplementary material for: Importance of congruence between communicating and executing implementation programmes: a qualitative study of focus group interviews
Source: Implement Sci Commun. 2020 Oct 28;1:94. doi: 10.1186/s43058-020-00090-w (PMC7594330; doi:10.1186/s43058-020-00090-w)
Supplement: Supplementary file 3 — Additional file 3. Descriptions and rationale behind of two instruments. [file 43058_2020_90_MOESM3_ESM.pdf]

DESCRIPTIONS AND RATIONALE BEHIND OF TWO INSTRUMENTS

Revised Socratic Approach for Health Technology Assessment

The Revised Socratic Approach for Health Technology Assessment is a comprehensive panel of questions intended to render a structured assessment of possible socioethical influences that the implementation of a health technology of interest may bring in or has already done in order to inform those responsible for decisions on them (1). It consists of seven basic questions, which are further broken down into thirty-three explanatory questions. The basic questions encompass the following domains related to the target technology: the target problem and group; ethical, cultural and societal challenges; challenges with structural changes; issues of characteristics; aspects of stakeholders; issues of the assessment itself; possible additional issues. It is aimed at eliciting reflexive dialogue between stakeholders and/or serving as a checklist for gathering relevant information. It is not an implementation theory or framework, nor a tool for performing a qualitative analysis.

According to World Health Organization, health technologies may be material or immaterial applications to solve health related problems (2). Thus, we deem the Revised Socratic Approach for Health Technology Assessment a relevant tool for gathering socioethical information on the influence of implementation programmes in health care context.

Normalization Process Theory

Normalization Process Theory (NPT) provides concepts for perceiving and explaining social processes intended to introduce new practices into routine use in the context of work (4,5). The term 'normalization' refers to a process through which a new practice is incorporated or institutionalized into a routine practice sustainably among the personnel of an organisation. It divides the process into three facets: implementing, embedding and integrating. Furthermore, NPT defines four mechanisms through which the process is operationalized, namely coherence, cognitive participation, collective action and reflexive monitoring. These are described in our coding frame for the qualitative content analysis in Table A, Additional file 5.

The NPT is aimed at being utilized in different phases and roles in implementing health technologies, implementation trials and evaluation of implementation programmes (5), which typically are complex processes in nature. We applied the NPT on building up the frame of coding and analysis as well as interpreting the findings.

References

1. Hofmann B, Droste S, Oortwijn W, Cleemput I, Sacchini D. Harmonization of ethics in health technology assessment: A revision of the socratic approach. Int J Technol Assess Health Care. 2014;30(1):3–9.
2. World Health Organization web page [Internet]. [cited 2020 Apr 28]. Available from: https://www.who.int/health-technology-assessment/about/healthtechnology/en/
3. Beebe J. Rapid Assessment Process. In: Kempf-Leonard K, editor. Encyclopedia of Social Measurement. New York: Elsevier; 2005. p. 285–91.
4. May C, Finch T. Implementing, embedding and integrating practices: an outline of Normalization Process Theory. Sociology. 2009;43(3):535–554.
5. May C, Rapley T, Mair FS, Treweek S, Murray E, Ballini L, et al. Normalization Process Theory On-line Users' Manual, Toolkit and NoMAD instrument. [Internet]. 2015. Available from: http://www.normalizationprocess.org/
