# Supplementary material for: Importance of congruence between communicating and executing implementation programmes: a qualitative study of focus group interviews
Source: Implement Sci Commun. 2020 Oct 28;1:94. doi: 10.1186/s43058-020-00090-w (PMC7594330; doi:10.1186/s43058-020-00090-w)
Supplement: Supplementary file 4 — Additional file 4. The interview guide for the first interviews of Focus Groups 1 and 2 and the process of deriving of the guide (Table A). The interview guide for the second interview of Focus Group 1 (Table B). [file 43058_2020_90_MOESM4_ESM.pdf]

Table A. Formulation of the interview guide for the first interviews of focus groups 1 and 2.

**Topic**	**Original basic question of Revised Socratic Approach**	**Original explanatory question of Revised Socratic Approach**	**Reformulated guiding question for the present study**	***Additional guidance for the facilitator***
Motives and reasoning behind the programme	5. What are the moral issues related to stakeholders?	Q21 What are the interests of the producers of technology (industry, universities)?	Q21 Why did the programme launchers', ultimately, want to carry out the programme? What were the fundamental motives and ambitions underlying the programme?	*Identify possible connections to interviewees' professional and the organisation's values?*
	6. What are the moral issues related to the assessment of the health technology?	Q30 Are there related or analogous technologies that have not been assessed? (Why not?)	Q30 Would there have been alternative ways to achieve the objectives set for the programme? If so, were they considered? Why were they set aside?	*Identify possible references to the organisation's Administrative Regulations, the Strategy and the Handbook of Management.*
Management of the programme	2. What are the ethical, social, cultural, legal and religious challenges related to the health technology?	Q6 Does the technology challenge social or cultural values, institutions, or arrangements or does it affect religious convictions?	Q6 How well does the operational realisation of the programme match the organisation's values and normal managerial practices?	*Review the structure of the managerial line organization.*

Case study of ODP_Lindholm et al.
Additional File 4

(Continues) Management of the programme	3. What are the moral challenges with structural changes related to the health technology?	Q12 Does the technology in any way challenge or change the relationship between patients and health care professionals or between health professionals?	Q12 Does the way of executing the programme put the personal relationships to the test within the managerial line? Or what about the personal relationships inside the involved teams?	*Review the factual realisation of the programme (designing process, programme plan, recruiting the teams, operational management ect.). Promote the conversation about these issues.*
	4. What are the moral issues related to the characteristics of the health technology?	Q15 Is the symbolic value of the technology of any moral relevance? (Prestige, status?) May this change as a result of the health technology?	Q15 Did the way of realising the programme comply with the regular managerial practices? Did the way of realising the programme somehow affect the performance of the managerial line, or, conversely,, did some phenomena within the managerial line affect the realisation of the programme?	
Perspective of the participating units	2. What are the ethical, social, cultural, legal, and religious challenges related to the health technology?	Q8 What are the morally relevant *consequences* (benefits and harms) of the implementation, use or withdrawal of the technology? (In particular from a patients' perspective). How should the harms be balanced against the benefits? Are there alternatives?	Q8 What possible positive or negative consequences has the way of realising the programme yielded in the involved teams (looked from their point of view)? What measures could have amplified the positive impacts and reduced the negative ones? Would there have been alternative ways to operate?	
	5. What are the moral issues related to stakeholders?	Q20 How does the technology contribute to or challenge or alter health professional's autonomy?	Q20 How did the way of realising the programme possibly affect the autonomy of the personnel involved? Increased, decreased or altered some other way? Or what about the autonomy of the units involved?	

Interest of the evaluators	6. What are the moral issues related to the assessment of the health technology?	Q28 What are the interests of the people participating in the technology assessment?	Q28 Why is the programme evaluated? What evaluation related interests do the members of the present focus group have?	
Quick vision on future developments			Vision on future developments.	*Ask the group to imagine the status of the topic after about 1-5 years. This theme is weighed more at the second interview of focus group 2.*

Table B. The interview guide for the second interview of Focus Group 1 (FG1).

**Part**	**Course of the interview**	**Guidance for the facilitator**	***Additional guidance***
A	Reviewing of the report on FGI1.1<br>↓<br>Reflective conversation about the report	Read the report section by section and elicit conversation, finding successes and troubles.	*During their second interview the FG1 is gradually directed to review both previous interview reports (FGI1.1 and FGI2) in an integrative way and infer possible future implications.*<br>*The idea is to involve the FG1 with the analysis of ODP processes instead of being only a source of data collection.*
B	Introducing the report on FGI2<br>↓<br>Reflective conversation about the report	Read the report section by section and elicit conversation, finding successes and problems.	
C	Scrutinizing reports on FGI1.1 and FGI2 together<br>↓<br>What future implications can be inferred?	What do these two reports tell us about the realization of the ODP? Invite the group to elaborate on measures for making the future developments better.	

FGI1.1 = first interview with FG1; FGI2 = interview with Focus Group 2; ODP = Ostrobothnia Depression Programme.
