# Supplementary material for: Importance of congruence between communicating and executing implementation programmes: a qualitative study of focus group interviews
Source: Implement Sci Commun. 2020 Oct 28;1:94. doi: 10.1186/s43058-020-00090-w (PMC7594330; doi:10.1186/s43058-020-00090-w)
Supplement: Supplementary file 5 — Additional file 5. Coding frame according to Normalization Process Theory. [file 43058_2020_90_MOESM5_ESM.pdf]

Table. Coding frame according to Normalization Process Theory (NPT)*.

**Main category (core construct of NPT)**	**Subcategory (component of core construct of NPT)**
**Coherence** is the sense-making work that people do individually and collectively when they are faced with the problem of operationalizing some set of practices.	**Differentiation**<br>Sense-making work is to understand how a set of practices and their objects are different from each other.<br><br>**Communal specification**<br>This refers to how people working together build a shared understanding of the aims, objectives and expected benefits of a set of practices.<br><br>**Individual specification**<br>This refers to need for participants to do things that will help them understand their specific tasks and responsibilities around a set of practices.<br><br>**Internalization**<br>This refers to efforts people make to understand the value, benefits and importance of a set of practices.
**Cognitive participation** is the relational work that people do to build and sustain a community of practice around a new technology or complex intervention.	**Initiation**<br>When a set of practices is new or modified, a core problem is whether or not key participants are working to drive them forward.<br><br>**Enrolment**<br>Participants may need to organize or reorganize themselves and others in order to collectively contribute to the work involved in new practices.<br><br>**Legitimation**<br>An important component of relational work around participation is the work of ensuring that other participants believe it is right for them to be involved, and that they can make a valid contribution to it.<br><br>**Activation**<br>Once it is underway, participants need to collectively define the actions and procedures needed to sustain a practice and to stay involved.

**Collective action** is the operational work that people do to enact a set of practices, whether these represent a new technology or complex healthcare intervention.	**Interactional workability**<br>This refers to the interactional work that people do with each other, with artefacts, and with other elements of a set of practices when they seek to operationalize them in everyday settings.<br><br>**Relational integration**<br>This refers to the knowledge work that people do to build accountability and maintain confidence in a set of practices and in each other as they use them.<br><br>**Skill set workability**<br>This refers to the allocation work that underpins the division of labour that is built up around a set of practices as they are operationalized in the real world.<br><br>**Contextual integration**<br>This refers to the resource work - managing a set of practices through the allocation of different kinds of resources and the execution of protocols, policies and procedures.
**Reflexive monitoring** is the appraisal work that people do to assess and understand the ways that a new set of practices affects them and others around them.	**Systematization**<br>Participants in any set of practices may seek to determine how effective and useful it is for them and for others, and this involves the work of collecting information in a variety of ways.<br><br>**Communal appraisal**<br>Participants work together - sometimes in formal collaboratives, sometimes in informal groups to evaluate the worth of a set of practices. They may use many different means to do this drawing on a variety of experiential and systematized information.<br><br>**Individual appraisal**<br>Participants in a new set of practices also work experientially as individuals to appraise its effects on them and the contexts in which they are set. From this work stem actions through which individuals express their personal relationships to new technologies or complex interventions.<br><br>**Reconfiguration**<br>Appraisal work by individuals or groups may lead to attempts to redefine procedures or modify practices - and even to change the shape of a new technology itself.

* http://www.normalizationprocess.org (read 7th June 2017).
